# Immunoglobulin a vasculitis with testicular/epididymal involvement in children: A retrospective study of a ten-year period

**DOI:** 10.3389/fped.2023.1141118

**Published:** 2023-03-20

**Authors:** Jian-Jun Hu, Yao-Wang Zhao, Rong Wen, Yang-Yang Luo, Wei-Guo Zhou, Yu-Hang Liu, Feng Qin, Chang Liu, Tian-Qu He

**Affiliations:** ^1^ Department of Urology, Hunan Children’s Hospital, Changsha, China; ^2^ Department of Pathology, Hunan Children’s Hospital, Changsha, China; ^3^ Department of Dermatology, Hunan Children's Hospital, Changsha, China; ^4^ Department of Urology, Ningyuan County People's Hospital, Yongzhou, China; ^5^ Academy of Pediatrics, Hengyang Medical School, University of South China, Hengyang, China

**Keywords:** igA vasculitis, henoch-Schönlein purpura, testicular/epididymal involvement, acute scrotum, acute epididymitis-orchitis

## Abstract

The clinical characteristics and risk factors for testicular/epididymal involvement in 73 children with immunoglobulin A vasculitis (IgAV) who were admitted to our hospital between January 2012 and November 2022 were reviewed. The demographic data, laboratory parameters, and follow-up data of the patients were compared to those of 146 males without testicular/epididymal involvement. A logistic regression analysis was performed to determine the variables associated with testicular/epididymal involvement. The prevalence of testicular/epididymal involvement among male patients with IgAV was 1.3% (73/5,556). Increased blood flow in the testes and/or epididymis on ultrasound was found in 71 patients. The remaining two patients underwent surgical exploration for loss or reduction of testicular blood flow. One patient underwent orchiectomy for intraoperative confirmation of complete right testicular infarction. Pathological findings revealed IgA immune complex deposition in the testis. Patient age (odds ratio [OR] = 0.792; 95% confidence interval [CI]: 0.682–0.919, *p* = 0.002), platelet count (OR = 1.011; 95% CI: 1.002–1.020, *p* = 0.013), and immunoglobulin M (IgM) levels (OR = 0.236; 95% CI: 0.091–0.608, *p* = 0.003) were strongly associated with the occurrence of testicular/epididymal involvement in IgAV. Therefore, young age, increased platelet count, and low IgM levels in patients with IgAV are potential risk factors for testicular/epididymal involvement. Doppler ultrasound can help differentiate IgAV from acute scrotum. Most patients with testicular/epididymal involvement have good prognoses, although serious complications such as testicular infarction may occur.

## Introduction

Immunoglobulin A (IgA) vasculitis (IgAV), also termed Henoch-Schönlein purpura, is the most common form of systemic vasculitis in the pediatric population (1). The pathogenesis of IgAV is unclear and may be related to infection, vaccination, food or drug factors, or genetics (2, 3). Pathologically, IgAV is characterized by leukocytoclastic vasculitis involving small vessels, predominately due to IgA immune complex deposition (3). IgAV is mostly self-limiting, and most patients have a good prognosis, although severe renal, gastrointestinal, and other organ damage may occur (3–5).

Scrotal involvement in patients with IgAV is rare. Clinical symptoms of scrotal involvement in IgAV include redness, swelling, and pain in the scrotum (6–8). Some patients exhibit enlargement of the testes and/or epididymis and increased blood flow on ultrasound (6–8). Rarely, patients have symptoms mimicking testicular torsion, requiring surgical exploration (9–11). Zhao et al*.* (12) reported the deposition of immune complexes containing IgA in affected testicular tissue, suggesting that the testis is a target organ for IgAV.

Laboratory parameters can be used as predictors of organ involvement in IgAV. The white blood cell count (WBC), neutrophil count (NEU), lymphocyte count (LYM), platelet count (PLT), neutrophil/lymphocyte ratio (NLR), and platelet/lymphocyte ratio (PLR) are associated with organ involvement in patients with IgAV (13–18). Buscatti et al*.* (7) reported that patients with IgAV and Scrotal involvement are less likely to have elevated serum IgA compared to patients with IgAV without Scrotal involvement. Their cohort included patients with simple scrotal purpura or edema. However, scrotal purpura and edema may be considered dermatological manifestations of IgAV. More serious complications, such as testicular infarction, may develop if the testes and/or epididymis are involved. To date, no controlled studies regarding the risk factors for testicular/epididymal involvement in patients with IgAV have been reported.

In this study, we used ultrasound to identify abnormalities of the testes and/or epididymis. A retrospective analysis of patients with IgAV with testicular/epididymal involvement who were admitted to our institution during the past ten years was conducted. Male patients with IgAV without testicular/epididymal involvement were used as a control group. This study investigated the clinical characteristics and associated risk factors for testicular/epididymal involvement in patients with IgAV.

## Materials and methods

### Patients

Between January 2012 and November 2022, 9,523 pediatric patients with IgAV were admitted to our hospital, including 73 with testicular/epididymal involvement. Male patients without testicular/epididymal involvement were randomly selected using the random number table method at a ratio of 2:1 to serve as the control group. The ethical committee of Hunan Children's Hospital approved this study (approval number: HCHLL-2022-156), and the requirement of informed consent was waived.

### Definitions

Patients were diagnosed with IgAV according to the joint classification criteria by the European League Against Rheumatism, Pediatric Rheumatology International Trials Organization, and Pediatric Rheumatology European Society (EULAR/PRINTO/PRES) (19). The presence of purpura or petechiae (mandatory) with lower limb predominance and at least one of the following four criteria are required for a diagnosis of pediatric IgAV: abdominal pain, histopathology, arthritis or arthralgia, or renal involvement (19).

In this study, gastrointestinal involvement of IgAV was defined as abdominal pain, vomiting, or gastrointestinal bleeding (15). Arthritis was defined as the acute onset of joint swelling or pain with limitation of motion, and arthralgia was defined as the acute onset of joint pain without joint swelling or limitation of motion (19). Renal involvement was defined as hematuria (> 5 red blood cells/high power field) or proteinuria (>150 mg/24 h) (20). Testicular/epididymal involvement was defined as the presence of abnormalities in the testes and/or epididymis on color Doppler ultrasound. Patients with only scrotal pain, scrotal edema, or purpuric rash were excluded from the study. Purpura recurrence was defined as the appearance of new purpura or petechiae after complete recovery.

### Data collection

Patient data were collected *via* the electronic medical record system. Patient age, chief complaint, and clinical presentation were collected as baseline data. Prior to treatment, the complete blood count (WBC, NEU, LYM, and PLT), mean platelet volume (MPV), hemoglobin, C-reactive protein, erythrocyte sedimentation rate, antistreptolysin O, immunoglobulin (Ig)A, IgE, IgG, IgM, and complement component 3 were measured. Imaging findings, pathological findings, follow-up duration, and follow-up outcomes were also recorded. The NLR was calculated by dividing the NEU by the LYM. The PLR was calculated by dividing the PLT by the LYM. The MPV/platelet count ratio (MPR) was calculated by dividing the MPV by the PLT.

Scrotal ultrasound was not routinely performed and was only conducted when a patient presented with symptoms localized to the scrotum (pain, purpura, or edema). Surgical exploration was performed in patients whose ultrasound results suggested decreased or absent testicular blood flow. During surgery, orchiectomy was performed if the testis was completely necrotic (no fresh blood flow upon incision of the tunica albuginea). When orchiectomy was not conducted, a small amount of testicular tissue was sampled for routine pathological examination, including hematoxylin and eosin staining and immunohistochemistry. Immunohistochemical staining was performed using IgA antibodies. Patients with testicular/epididymal involvement underwent dynamic ultrasound during the treatment period.

### Statistics

The Shapiro–Wilk test was used to test the normality of the data. Continuous data with a normal distribution are presented as mean ± standard deviation and those without a normal distribution are presented as median and interquartile range (Q1–Q3). Categorical data are presented as numbers and frequencies. The *t*-test was used to compare the normally distributed continuous data between the two groups. The rank-sum test was used to compare data without a normal distribution. The chi-square test was used to compare the categorical data of the two groups. Variables with a *p* value < 0.2 in the univariate analysis were included in the multivariate logistic regression analysis. All statistical analyses were conducted using SPSS version 25.0 software (IBM, USA). Statistical significance was set at *p *< 0.05.

## Results

The mean age at presentation was 7.3 years (range: 5.5–9.5 years), and 3,967 (41.7%) female patients and 5,556 (58.3%) male patients were treated. Testicular/epididymal involvement was diagnosed in 73 patients (1.3% of male patients). The control group included 146 male patients.

Purpura was listed as the first symptom in 44 patients (60.3%) in the testicular/epididymal involvement group. Abdominal pain was the first symptoms in 19 patients (26.0%), and arthralgia in 10 patients (13.7%). The average time from the first symptom to the appearance of scrotal symptoms was 9.0 days (range: 5.0–15.0 days). The average duration of scrotal symptoms was 5.0 days (range: 4.0–6.0 days). The average scrotal wall thickness on ultrasound was 6.0 mm (range: 3.6–8.0 mm).

**Figure 1:**
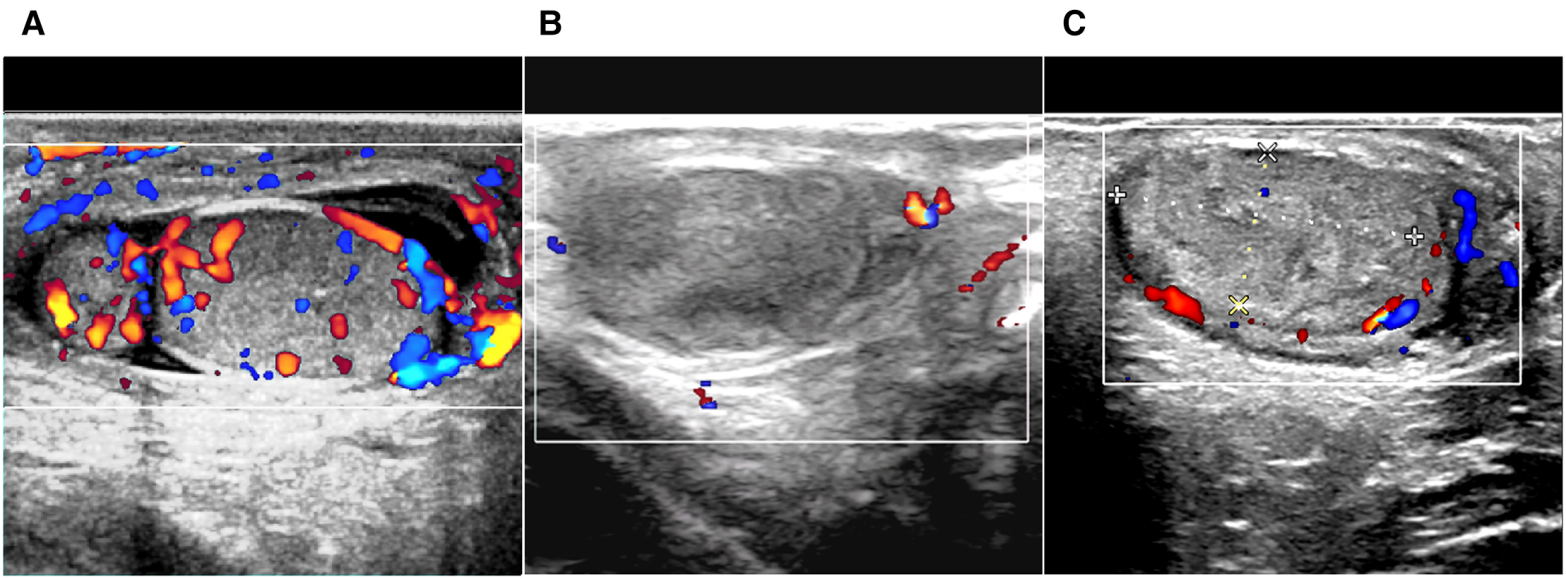
Ultrasound scan of immunoglobulin A vasculitis with testicular/epididymal involvement. (**A**) Enlarged epididymis, fluid retention in the scrotum, and increased blood flow in the testis are shown on color Doppler. (**B,C**) The testis has hyperechoic and heterogeneous echoic areas, and the intratesticular blood flow is absent (**B**) or decreased (**C**).

Twenty-four patients had bilateral involvement, 24 had right-side only involvement, and 25 had left-side only involvement. Of the 73 patients with testicular/epididymal involvement, 71 had an enlarged testis and/or epididymis with abundant blood flow on ultrasound (Figure 1A). The remaining two patients showed loss or reduction of testicular blood flow.

**Figure 2:**
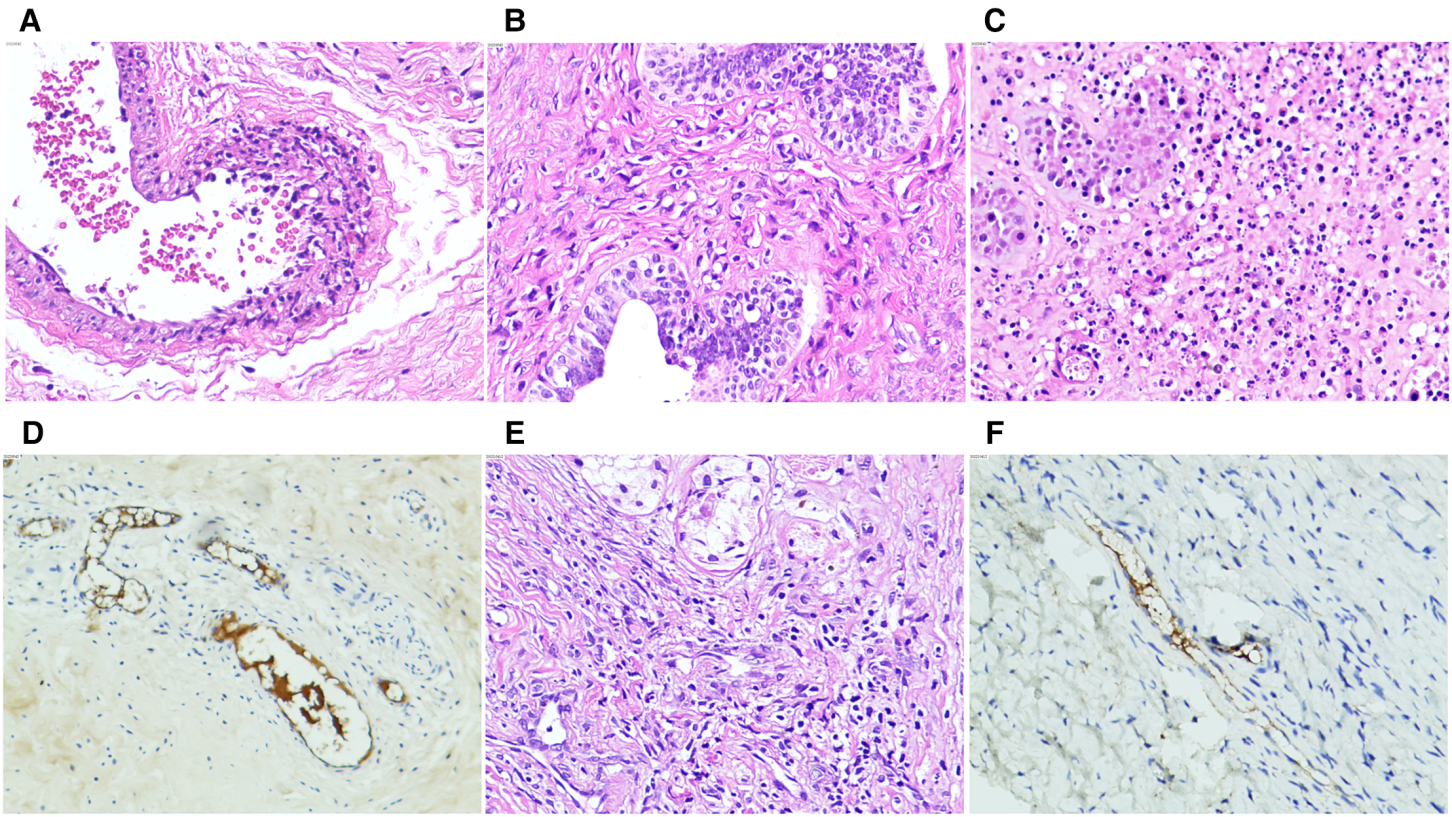
Photomicrographs of immunoglobulin A vasculitis with testicular/epididymal involvement. (**A–D**) A four-year-old patient underwent orchiectomy for complete right testicular infarction. (**A**) Neutrophil infiltration with localized necrosis is shown in the vascular wall of the spermatic cord (hematoxylin and eosin (H&E, ×20). (**B**) Lymphocytic infiltration is shown in the wall of the vas deferens (H&E, ×20). (**C**) Most of the germinal tubules are necrotic with neutrophil, lymphocyte, and monocyte infiltration (H&E, ×20). (**D**) Immunohistochemical stains showing immunoglobulin A immune complex deposits are observed in the vascular walls of the epididymis and testicular interstitium at several sites. (**E,F**) Partial infarction of the right testicle of a six-year-old patient is shown. (**E**) HE staining shows the testicular parenchyma and interstitial necrosis with lymphocytic infiltration in the vessel wall (×20). (**F**) Immunohistochemical staining shows immunoglobulin A immune complex deposition in the vascular wall of the testicular tissue (×20).

All patients with testicular/epididymal involvement were treated with steroids. Two patients underwent surgical exploration. Of the two patients, one was admitted for ten days with a rash and abdominal pain and developed scrotal symptoms on the third day of treatment. An immediate ultrasound examination revealed no significant blood flow in the right testicle (Figure 1B). Subsequent surgical exploration revealed complete infarction of the right testicle, and an orchiectomy was performed. The second patient presented with scrotal symptoms on the fourth day after discharge from the hospital with recovery from IgA vasculitis. A mixed echogenic mass was detected in the right testis with reduced blood flow on ultrasound (Figure 1C). Conservative treatment with antibiotics and steroids was administered for two weeks. Serial ultrasound of the right testis showed no significant changes in echogenicity and blood flow. A surgical exploration was subsequently performed. Upon incision of the tunica albuginea, only a small amount of fresh blood flow was detected. The immunohistochemical results of these two patients revealed IgA immune complex deposition in the testis (Figure 2).

The median age of the patients with testicular/epididymal involvement was 5.4 years and that of patients without testicular/epididymal involvement was 7.2 years (*p *< 0.001). There were no differences regarding the involvement of other systems or organs between the groups. The median PLT was higher among patients with testicular/epididymal involvement (380.0 × 10^3^/*μ*l vs. 322.0 × 10^3^/μl, *p *< 0.001), whereas the MPR (0.026 vs. 0.030, *p *= 0.001) and IgM levels (0.9 g/L vs. 1.1 g/L, *p *< 0.001) were lower in patients with testicular/epididymal involvement. The follow-up duration and purpura recurrence rate were not significantly different between the groups (Table 1).

**Table 1:** Comparison of clinical features and laboratory and follow-up data of the two groups.

	Testicular/epididymal involvement group (*n* = 73)	Control group (*n* = 146)	*t*/*z*/χ2	*P*
Age (years)	5.4 (4.0–7.2)	7.2 (5.5–9.6)	−4.805	<0.001
Renal involvement, *n* (%)	21 (28.8)	38 (26.0)	0.186	0.667
GI involvement, *n* (%)	45 (61.6)	76 (52.1)	1.810	0.179
Joint involvement, *n* (%)	50 (68.5)	91 (62.3)	0.806	0.369
WBC (10^3^/μl)	11.2 (9.1–13.4)	9.6 (7.9–13.0)	−1.874	0.061
Neutrophil (10^3^/μl)	7.1 (5.3–9.4)	6.3 (4.4–9.1)	−1.518	0.129
Lymphocyte (10^3^/μl)	3.0 (2.2–3.9)	2.6 (2.0–3.5)	−1.411	0.158
Platelet (10^3^/μl)	380.0 (314.5–490.0)	322.0 (276.5–383.5)	−4.133	<0.001
MPV (fl)	9.7 (9.0–10.7)	9.8 (9.1–10.3)	−0.427	0.670
HGB (g/l)	121.1 ± 14.8	124.4 ± 15.1	0.106	0.122
NLP	2.6 (1.5-3.6)	2.3 (1.3-3.8)	−0.745	0.456
PLR	128.8 (96.1–198.0)	117.1 (87.0–173.7)	−1.442	0.149
MPR	0.026 (0.019–0.032)	0.030 (0.024–0.037)	−3.211	0.001
CRP (mg/L)	7.9 (2.8–12.1)	4.3 (1.8–11.4)	−1.937	0.053
ESR (mm/h)	8.0 (3.5–12.5)	7.0 (2.0–12.0)	−0.877	0.381
ASO (IU/ml)	25.0 (15.7–109.8)	25.0 (20.4–84.4)	−0.040	0.968
IgA (g/L)	2.2 (1.5–2.7)	2.1 (1.5–2.8)	−0.395	0.693
IgG (g/L)	9.1 (6.9–11.0)	9.3 (7.7–11.6)	−1.485	0.137
IgM (g/L)	0.9 (0.7–1.1)	1.1 (0.8–1.5)	−4.577	<0.001
IgE (IU/ml)	62.5 (25.8–180.2)	70.7 (26.8–180.3)	−0.425	0.671
C3 (g/L)	1.1 (0.9–1.2)	1.1 (0.9–1.1)	−0.364	0.716
Follow up period (month)	20.0 (12.0–36.0)	19.5 (12.0–28.0)	−0.822	0.411
Recurrent purpura, *n* (%)	17 (23.3)	26 (17.8)	0.926	0.336

GI, gastrointestinal; WBC, white blood cell; MPV, mean platelet volume; NLR, blood neutrophil-to-lymphocyte ratio; PLR, blood platelet-to-lymphocyte ratio; MPR, MPV /platelet count ratio; CRP, C-reactive protein; ESR, erythrocyte sedimentation rate; ASO, Antistreptolysin O; Ig, immunoglobulin; C3, complement 3.

Patient age (odds ratio [OR] = 0.792; 95% confidence interval [CI]: 0.682–0.919, *p* = 0.002), PLT (OR = 1.011; 95% CI: 1.002–1.020, *p* = 0.013), and IgM levels (OR = 0.236; 95% CI: 0.091–0.608, *p* = 0.003) were independently associated with testicular/epididymal involvement (Table 2).

**Table 2:** Logistic regression analysis of predictors for testicular/epididymal involvement in IgAV patients.

	B	S.E	Wald	*P*	OR	95% CI
Age	−0.234	0.076	9.378	0.002	0.792	0.682–0.919
Platelet count	0.011	0.005	6.107	0.013	1.011	1.002–1.020
IgM level	-1.445	0.483	8.942	0.003	0.236	0.091–0.608

IgAV, Immunoglobulin A vasculitis; CI, confidence interval; OR, odds ratio; Ig, immunoglobulin.

## Discussion

This study reports the largest cohort of pediatric patients with IgAV with testicular/epididymal involvement to date. The prevalence of scrotal involvement in patients with IgAV has been reported to be 2%–38%; this large variability is due to the different definitions of scrotal involvement used in previous studies (6–8, 21–24). In this study, a simple purpura or edema was considered a dermatological manifestation of IgAV, as genital involvement was believed to result in more serious complications. We defined testicular/epididymal as abnormalities of the testes and/or epididymis identified on ultrasound. Testicular/epididymal involvement was diagnosed in 1.3% of the male patients in this study.

Color ultrasound is useful for the differential diagnosis of acute scrotum (6, 22, 23, 25). In this study, 97.3% (71/73) of patients with testicular/epididymal involvement had increased blood flow in the testes and/or epididymis on ultrasound (Figure 1A). The remaining two patients underwent surgical exploration, and no testicular torsion was detected. The ultrasound findings of the patients who underwent surgical exploration indicated the loss or reduction of intra-testicular blood flow and heterogeneous testicular echogenicity with hyperechoic areas, which are typically attributed to ischemia and infarction (Figures 1B,C) (25). The immunohistochemical findings of these two patients revealed IgA immune complex deposition in the testis (Figure 2), confirming that the testis is a target organ of IgAV, as previously reported by Zhao et al*.* (12).

Only one patient with IgAV combined with testicular torsion has been reported (9). Ma et al*.* (26) reviewed the cases of 21 patients with IgAV with scrotal involvement published between 1986 and 2020, including five who underwent surgical exploration. No patient in the previous review was diagnosed with testicular torsion. Testicular infarction due to IgAV mimics the presentation of testicular torsion on ultrasound, and surgical exploration is safe and justified to diagnose testicular torsion in these patients (22, 27). However, when mechanical etiologies of testicular torsion are excluded intraoperatively, there is no surgical treatment for testicular infarction secondary to IgAV.

In this study, the conservative treatment of testicular epididymitis associated with IgAV was successful, as no patients had recurrence at follow-up. Previous studies have recommended steroids for the treatment of testicular epididymitis associated with IgAV (6–8); however, long-term fertility remains a concern.

A number of studies reported no significant differences regarding the involvement of other systems or organs between patients with scrotal involvement and those without scrotal involvement (6, 7). Similarly, in the present study, gastrointestinal tract, joint, and renal involvement were not significantly different between the two groups. These results suggest no association between testicular/epididymal involvement and the involvement of other systems.

In this study, young age, elevated platelet count, and lower IgM level were identified as risk factors for testicular/epididymal involvement. In a previous study by Coşkun et al*.* (28), 19/117 (16.2%) preadolescent male patients had scrotal involvement, and 6/70 (8.5%) adolescent male patients had scrotal involvement. Sunar et al*.* (6) reported that scrotal involvement occurred significantly more frequently in younger patients, which is consistent with the results of the current study. Several studies have reported an age variability regarding gastrointestinal and renal involvement in patients with IgAV (29).

A recent study reported that thrombocytosis in children with IgAV is a type of inflammatory reactive thrombocytosis (30). During the acute phase of IgAV, cytokines such as tumor necrosis factor-α, interleukin-6, and interleukin-8 are significantly increased, leading to thrombocytosis and a more severe disease course (31, 32). Elmas et al*.* (17) reported that thrombocytosis is an independent risk factor for renal involvement in patients with IgAV. In the present study, PLT count was strongly associated with testicular/epididymal involvement. It was hypothesized that thrombocytosis promotes thrombosis during the course of IgAV and may contribute to testicular infarction. In addition, deep vein thrombosis and venous sinus thrombosis secondary to IgAV have been reported (33, 34). Although the significance of IgM levels in patients with IgAV remains unclear, the results of this study suggest that IgM levels are closely associated with testicular/epididymal involvement. Further studies are necessary to determine the mechanism of action.

This study has some limitations. First, it was a retrospective study that did not control for exposure and interventions, leading to potential confounding bias. Second, the single-center study had a limited sample size and regional and environmental biases. Multi-center prospective studies are needed to confirm the results of this study.

In conclusion, young age, increased PLT, and low IgM levels are potential risk factors for testicular/epididymal involvement involvement in patients with IgAV. Color Doppler ultrasound and a history of IgAV can help differentiate testicular/epididymal involvement of IgAV from acute scrotum. Most patients with testicular/epididymal involvement have a good prognosis, although serious complications, such as testicular, infarction may occur.

## Data Availability

The raw data supporting the conclusions of this article will be made available by the authors, without undue reservation.

## References

[1] Ozen S Sag E. Childhood vasculitis. Rheumatology (Oxford). (2020) 59:i95–100. 10.1093/rheumatology/kez599 32348513

[2] Oni L Sampath S. Childhood IgA vasculitis (henoch schonlein purpura)—advances and knowledge gaps. Front Pediatr. (2019) 7:257. 10.3389/fped.2019.00257 31316952 PMC6610473

[3] Song Y Huang X Yu G Qiao J Cheng J Wu J Pathogenesis of IgA vasculitis: an up-to-date review. Front Immunol. (2021) 12:771619. 10.3389/fimmu.2021.771619 PMC8630619 34858429

[4] Xu L Li Y Wu X. IgA vasculitis update: epidemiology, pathogenesis, and biomarkers. Front Immunol. (2022) 13. 10.3389/fimmu.2022.921864 PMC9574357 36263029

[5] Pillebout E Sunderkötter C. IgA vasculitis. Semin Immunopathol. (2021) 43:729–38. 10.1007/s00281-021-00874-9 34170395

[6] Sunar YE Bakkaloglu SA. Clinical features of pediatric IgA vasculitis patients with scrotal involvement. Mod Rheumatol. (2022) 15:roac111. 10.1093/mr/roac111 36107741

[7] Buscatti IM Abrão HM Kozu K Marques VLS Gomes RC Sallum AME Characterization of scrotal involvement in children and adolescents with IgA vasculitis. Adv Rheumatol. (2018) 58:38. 10.1186/s42358-018-0039-3 30657092

[8] Ha TS Lee JS. Scrotal involvement in childhood henoch-schönlein purpura. Acta Paediatr. (2007) 96:552–5. 10.1111/j.1651-2227.2006.00173.x 17306010

[9] Kajitani S Miyamoto M Tokura Y Mizuno T Kambara T Ichikawa G Testicular torsion associated with henoch-schönlein purpura. J Pediatr. (2022) 243:231–2. 10.1016/j.jpeds.2021.12.001 34890587

[10] Akgun C. A case of scrotal swelling mimicking testicular torsion preceding henoch-schönlein vasculitis. Bratisl Lek Listy. (2012) 113:382–3. 10.4149/BLL_2012_087 22693978

[11] Dayanır Y Akdilli A Karaman C Sönmez F Karaman G. Epididymoorchitis mimicking testicular torsion in henoch-schönlein purpura. Eur Radiol. (2001) 11:2267–9. 10.1007/s003300100843 11702171

[12] Zhao L Zheng S Ma X Yan W. Henoch-schönlein purpura with testicular necrosis: sonographic findings at the onset, during treatment, and at follow-up. Urology. (2017) 107:223–5. 10.1016/j.urology.2017.05.005 28499760

[13] Yang Y Shu J Mu J He Q Chen F Hu Y Clinical analysis of 99 children with henoch-schönlein purpura complicated with overt gastrointestinal bleeding. Clin Rheumatol. (2022) 41:3783–90. 10.1007/s10067-022-06323-8 35941339

[14] Karadağ ŞG Çakmak F Çil B Tanatar A Sönmez HE Kıyak A The relevance of practical laboratory markers in predicting gastrointestinal and renal involvement in children with henoch-schönlein purpura. Postgrad Med. (2021) 133:272–7. 10.1080/00325481.2020.1807161 32772751

[15] Özdemir ZC Çetin N Kar YD Öcal HO Bilgin M Bör Ö. Hemotologic indices for predicting internal organ involvement in henoch-schönlein purpura (IgA vasculitis). J Pediatr Hematol Oncol. (2020) 42:e46–9. 10.1097/MPH.0000000000001571 31851146

[16] Hong SH Kim CJ Yang EM. Neutrophil-to-lymphocyte ratio to predict gastrointestinal bleeding in henoch: schönlein purpura. Pediatr Int. (2018) 60:791–5. 10.1111/ped.13652 29947449

[17] Elmas AT Tabel Y. Platelet counts in children with henoch-schonlein purpura-relationship to renal involvement. J Clin Lab Anal. (2016) 30:71–4. 10.1002/jcla.21817 25385472 PMC6806727

[18] Fu W Ye W Liu X Zhu S Fu H Zhu R Meta-analysis of the neutrophil-to-lymphocyte and platelet-to-lymphocyte ratios in henoch-schonlein purpura and its complications. Int Immunopharmacol. (2021) 94:107454. 10.1016/j.intimp.2021.107454 33588173

[19] Ozen S Pistorio A Iusan SM Bakkaloglu A Herlin T Brik R Eular/printo/pres criteria for henoch-schönlein purpura, childhood polyarteritis nodosa, childhood wegener granulomatosis and childhood takayasu arteritis: ankara 2008. Part ii: final classification criteria. Ann Rheum Dis. (2010) 69:798–806. 10.1136/ard.2009.116657 20413568

[20] Liu C Luo L Fu M Li Z Liu J. Analysis of children with henoch–schonlein purpura secondary to infection. Clin Rheumatol. (2022) 41:803–10. 10.1007/s10067-021-06007-9 34993728

[21] Tabel Y Inanc FC Dogan DG Elmas AT. Clinical features of children with henoch-schonlein purpura: risk factors associated with renal involvement. Iran J Kidney Dis. (2012) 6:269–74. 10.1186/s42358-018-0039-3 22797096

[22] Ioannides AS Turnock R. An audit of the management of the acute scrotum in children with henoch-schonlein purpura. J R Coll Surg Edinb. (2001) 46:98–9. 11329751

[23] Ben-Sira L Laor T. Severe scrotal pain in boys with henoch-schönlein purpura: incidence and sonography. Pediatr Radiol. (2000) 30:125–8. 10.1007/s002470050029 10663526

[24] Allen DM Diamond LK Howell DA. Anaphylactoid purpura in children (schonlein-henoch syndrome): review with a follow-up of the renal complications. AMA J Dis Child. (1960) 99:833. 10.1001/archpedi.1960.02070030835021 13792721

[25] Deeg KH. Differential diagnosis of acute scrotum in childhood and adolescence with high-resolution duplex sonography. Ultraschall Med. (2021) 42:10–38. 10.1055/a-1325-1834 33530122

[26] Ma Y Zhang S Chen J Kong H Diao J. Henoch-schönlein purpura with scrotal involvement: a case report and literature review. J Pediatr Hematol Oncol. (2021) 43:211–5. 10.1097/MPH.0000000000002161 33885039 PMC8327932

[27] Hara Y Tajiri T Matsuura K Hasegawa A. Acute scrotum caused by henoch-schönlein purpura. Int J Urol. (2004) 11:578–80. 10.1111/j.1442-2042.2004.00835.x 15242376

[28] Coşkun S Güngörer V Ekici Tekin Z Çelikel E Kurt T Tekgöz N Preadolescent-versus adolescent-onsetigav: the impact of age on prognosis. Pediatr Int. (2022) 65:e15426. 10.1111/ped.15426 36416667

[29] Aziz DA Siddiqui F Siddiqui MT. Age related characteristics of children and adolescent with henoch schönlein purpura and systems involvement: an experience from tertiary care center. J Ayub Med Coll Abbottabad. (2022) 34:336–40. 10.55519/JAMC-02-9750 35576298

[30] Lin CY Yang YH Lee CC Huang CL Wang LC Chiang BL. Thrombopoietin and interleukin-6 levels in henoch-schönlein purpura. J Microbiol Immunol Infect. (2006) 39:4–7682. 17164950

[31] Gasparyan AY Ayvazyan L Mukanova U Yessirkepov M Kitas GD. The platelet-to-lymphocyte ratio as an inflammatory marker in rheumatic diseases. Ann Lab Med. (2019) 39:345–57. 10.3343/alm.2019.39.4.345 30809980 PMC6400713

[32] Kimura S Takeuchi S Soma Y Kawakami T. Raised serum levels of interleukins 6 and 8 and antiphospholipid antibodies in an adult patient with henoch-schönlein purpura. Clin Exp Dermatol. (2013) 38:730–6. 10.1111/ced.12089 24073654

[33] Zheng Q He Q Huang H Lu M. Venous sinus thrombosis in a case of immunoglobulin a vasculitis and a systemic review of literature. Int J Rheum Dis. (2022) 26:539–43. 10.1111/1756-185X.14529 36502505

[34] Zaidi AU Berman B. Crossing the thrombotic threshold. Clin Pediatr (Phila). (2014) 53:1396–8. 10.1177/0009922814526983 24634422

